# Hypomineralised second primary molars: prevalence, pattern and associated co morbidities in 8- to 10-year-old children in Ile-Ife, Nigeria

**DOI:** 10.1186/s12903-016-0225-9

**Published:** 2016-06-04

**Authors:** T. A. Oyedele, M. O. Folayan, E. O. Oziegbe

**Affiliations:** Department of Surgery, Benjamin Carson, Snr, School of Medicine, Babcock University, Ilisan-Remo, Ogun State Nigeria; Dental Department, Babcock University Teaching Hospial, Ilisan-Remo, Ogun State Nigeria; Department of Child Dental Health, Obafemi Awolowo University Teaching Hospitals Complex, Ile-Ife, Osun State Nigeria; Department of Child Dental Health, Faculty of Dentistry, Obafemi Awolowo University, Ile-Ife, Osun State Nigeria

**Keywords:** Primary teeth, Caries, Oral hygiene, HSPM, MIH

## Abstract

**Background:**

This study tries to determine the prevalence and co-morbidities associated with hypomineralised second primary molars (HSPM) in 8- to 10- year-old children in Ile-Ife, Nigeria; and the co-existence of HSPM and Molar Incisor Hypomineralisation (MIH) in the study population.

**Methods:**

This was a cross sectional study involving 8- to 10- year-old children in schooling in suburban Nigeria. Information was collected on the child's age, sex and socioeconomic status. Intraoral examination was conducted to determine the presence of HSPM, MIH, caries and the oral hygiene status of study participants. The severity of HSPM was also determined. The prevalence of HSPM, the association between HSPM, sex and socioeconomic status of study participants, the difference in the prevalence of caries and poor oral hygiene in children with and without HSPM, and the prevalence of HSPM and MIH co-morbidity were determined.

**Results:**

Twenty seven of the 469 children examined (5.8 %) had HSPM. The tooth prevalence of HSPM was 3.9 %. There was no significant sex (*p* = 0.06), age (*p* = 0.41), and socioeconomic status (*p* = 0.67) differences between children with HSPM and without HSPM. More children with HSPM had caries (p ≤ 0.001) and poor oral hygiene (*p* = 0.01). Children with HSPM have increased odds having dental caries (AOR: 6.34; CI: 2.78–14.46; *p* = <0.001) and reduced odds of having good oral hygiene (AOR: 0.32; CI: 0.13–0.78; *p* = 0.01) when compare with children without HSPM. Also 77.8 % of children with HSPM also had MIH.

**Conclusion:**

The prevalence of HSPM in the study population is significantly high. The large number of children with HSPM and MIH also suggests that HSPM is a predisposing factor for MIH. The significantly higher proportion of children with HSPM who had caries and poor oral hygiene makes it imperative to institute screening programmes for HSPM/MIH in the study population.

## Background

Hypomineralised second primary molar (HPSM), also known as deciduous molar hypomineralisation (DMH), results from the disruption of the mineralisation of the 37 enamel in the second primary molar(s) during its development [1–3]. It is a structural or qualitative defect of enamel identified visually as clearly demarcated area of alteration in 39 the translucency of the enamel. This alteration may be of varying degree resulting in white, yellow or brown colour of the enamel [4, 5]. Study reports on HSPM are fairly new. There are recent publications on guidelines to help standardize studies on HSPM and molar incisor hypomineralisation (MIH) [6, 7]. Prior reports had focused on the prevalence and pattern of presentation of MIH. MIH is defined as the clinical appearance or morphological enamel defects involving the occlusal and/or incisal third of one or more permanent molars or incisors as a result of hypomineralisation of systemic origin [8]. This enamel defect results from disturbances in the calcification process. HSPM is the hypomineralisation of the second primary molars [2, 3]. The calcification of the second primary molars begins at 4th month of fetal life and formation complete at 36th month. This is somewhat earlier than the development of the first permanent molars and incisors, but the periods of development of the second primary molars and the first permanent molars overlap [9, 10]. If the risk factors associated with hypomineralisation occur during this overlapping period, hypomineralisation might occur in both the primary and permanent dentition [11]. Clinically, the clinical feature of HSPM is like that of MIH: white, yellow or brown discolouration of the enamel [12, 13]. The developmental defect can create considerable discomfort to the child due to shooting pain arising from taking cold meals or even breathing cold air shortly after the eruption of the affected teeth has started, can give the parents concern, and can present management problems for clinicians [14]. The defective enamel can also be a locus of lowered resistance for caries because there is an increase in porosity and consistently disorganised rod structure in the hypomineralised teeth [14]. Co-morbidities such as caries, dentine sensitivity, and poor oral hygiene have also been associated with hypomineralisation [15]. Chronologically, the second primary molars erupt about a year after the first primary molars [1, 5], however higher prevalence 64 of dental caries have been observed in second primary molars compare to first primary molar that may be explained by the predilection of HSPM for the second primary molars [1, 16]. HSPM may therefore be a significant risk factor for early loss of the second primary molars since its prevalence range from 4.6 % to 6.6 % [13, 17, 18]. Presently there is little information about the prevalence and risk factors associated with HSPM. This study therefore aimed at determining the prevalence and co-morbidities associated with HSPM, and the prevalence of HSPM/MIH comorbidity.

## Methods

The methodology for this study had been reported earlier by Oyedele et al. [19]. This paper reports part of the outcome of a large cross sectional study conducted to determine the co-morbidities associated with MIH in pupils aged eight to ten years resident in Ife central Local Government of Ile-Ife, a suburban town in the South-Western region of Nigeria. Study participants were selected from schools through a multi-staged sampling technique that resulted in the selection of a representative sample of public and private schools in the town. Pupils whose legal guardian consented to their participation and those who gave assent to study participation were eligible to participate in the study. Children with hypodontia, anodontia and amelogenesis imperfecta were excluded from the study.

### Sample size

The sample size was determined by the statistical formula proposed by Araoye [20]. The estimated proportion of children with MIH was 40 %, using the highest reported prevalence from various studies reported [21]. The calculated sample size needed with 10 % attrition rate was 405 children. However, 469 children were examined.

### Sample selection

The study location, Ife Central Local Government was divided into three geographical areas, each consisting of four political wards. One political ward was selected from each geographical area. In each ward, the schools were stratified into public primary and private primary, and one school was randomly selected from each stratum, by balloting. All the six schools selected provided a complete list of the children in each class. All pupils between 8 and 10 years in the schools were provided with consent forms for their parents/guardians to fill.

### Data collection

An interviewer-administered questionnaire was used. The data collection tool captured details of the age and sex of the child, parents’ educational level and parents’ occupation. Socioeconomic status for the purpose of this study was determined through a multiple- item scoring index [22], which has been used in prior studies in Nigeria [23, 24]. The status is designated according to the mother’s level of education and the occupation of the father. Each child was allocated to a social class from I to V, with social class V being the lowest of the classes. Each child’s social class was classified as Class I (upper class), class II (upper middle class), class III (middle class), class IV (lower middle class), or class V (lower class). When a child had lost a parent or was living with others, the socioeconomic status of the parent of the others whom the child resides with was adopted as the child’s socioeconomic status.

### Clinical examination

Each child was examined by one of the authors (TAO) using natural light, while seated on the school chair. The children were first examined for the oral hygiene status before proceeding to examine for HSPM. For the HSPM, the teeth were examined wet. However, debris was removed with a gauze swab where present. A trained assistant helped to record findings during intra-oral examination.

#### Oral hygiene

Oral hygiene was recorded using the simplified-oral hygiene index (OHI-S) described by Greene and Vermillion [27]. The OHI-S has two components; the debris index and the calculus index. Each of these indexes in turn, is based on numerical determinations representing the amount of debris or calculus found on the preselected tooth surfaces.

The six surfaces examined for OHI-S are selected from four posterior and two anterior teeth (upper right first molar, upper right central incisor, upper left first molar, lower left central incisor, lower left first molar and lower right first molar). The labial surfaces of upper right first molar, upper right central incisor, upper left first molar and lower left central incisor were examined while the lingual surfaces of lower right and left first molars were examined. Each tooth surface was divided into gingival, middle and incisal thirds. The scoring was: score 0- no debris or stain present; score 1- soft debris covering not more than one-third of the tooth surface; score 2- soft debris covering not more than two-thirds of the exposed tooth surface; score 3-soft debris covering more than two- thirds of the exposed tooth surface. The debris index simplified score is the sum of the debris score for all teeth, divided by the number of the surfaces scored. For each child, the debris score was averaged across the six individual teeth in order to obtain a single score per child.

For the calculus index simplified, the same 6 surfaces were examined with score of 0 to 3 dividing each surface horizontally into gingival, middle and incisal thirds. score 0- no calculus present; score 1- calculus covering not more than one-third of the tooth surface; score 2- calculus covering not more than two-thirds of the exposed tooth surface; score 3-calculus covering more than two-thirds of the exposed tooth surface. The calculus index simplified score is the sum of the calculus score for all teeth, divided by the number of the surfaces scored. For each child, the calculus score was averaged across the 6 individual teeth in order to obtain a single score per child.

The calculus index simplified (CI-S) and debris index simplified (DI-S) value may range from 0 to 3, the OHI-S value which is the sum of CI-S and DI-S range from 0 to 6. Oral hygiene index score of 0–1.2 means good oral hygiene; 1.3–3.0 means fair oral hygiene and 3.0–6.0 means poor oral hygiene.

#### Diagnosis of HSPM

Children were diagnosed with HSPM based on the criteria used by Elfrink et al. [13] when at least one second primary molar was affected. All the surfaces of the primary second molars were examined for demarcated opacity on the occlusal and buccal part of the crown. The colour of the opacity may vary from white, creamy or yellow to brownish. The defect can be negligible or comprise the major part of the crown. Each child’s HSPM severity was defined by the most severe defect seen in the permanent first molars and primary second molars.

The severity of HSPM was determined using the criteria highlighted by Weerheijm et al. [5]. Mild lesions were those with demarcated opacities present in the non-stress bearing areas of the molars with no enamel loss from fracturing. Moderate lesions were those with presence of any atypical restoration, demarcated opacities present on the occlusal/incisal third of the teeth with no post eruptive enamel break down or with post- eruptive enamel breakdown limited to one or two surfaces without cuspal involvement.

Severe lesions were those with post eruptive enamel breakdown. Diagnosis of MIH: The diagnosis of MIH was made based on the EAPD diagnostic criteria described by Weerheijm et al. [5]. Children were diagnosed with MIH when at least one permanent first molar was affected, with or without the involvement of the incisors. All the surfaces of the permanent first molar and the central incisor were examined for defined opacity. Each child’s MIH severity was defined by the most severe defect seen in the permanent first molars or permanent incisors.

#### Diagnosis of caries

Caries diagnosis was based on the recommendations of World Health Organisation oral health survey methods [25]. The examination for dental caries was conducted with a plane mouth mirror, using the light source from a torch, while the child was seated on a chair. The examination was done in an orderly manner; from one tooth or tooth space, to the adjacent tooth or tooth space. Caries status was assessed using the decayed missing and filled (dmft) index. Decayed (d) teeth were defined as any tooth whose crown had an unmistakable cavitation on the pits or fissures, or on a tooth surface or a filled crown with decay, when it has one or more permanent restorations that are decayed. The filled (f) was defined as a filled crown with no decay, when it has one or more permanent restorations, and there is no caries anywhere on the crown. The missing (m) was defined as a missing tooth due to caries; when a tooth has been extracted due to caries. To arrive at a dmft score for an individual patient’s mouth, three values must be determined: the number of teeth with carious lesions, the number of extracted teeth due to caries, and the number of teeth with fillings or crowns [26]. The numbers of teeth were then summed together to give the dmft score. Further questions were asked where there was a missing primary second molar and/or permanent first molar to determine the reason for the extraction.

### Data analysis

Descriptive analysis was used to describe the demographic variables (age, sex and socio- economic status), the prevalence and severity of HPSM. Pearson’s Chi-square test was used to establish the association between HPSM, age, sex and socio-economic status. A t- test was also conducted to determine the difference in the dmft of children with and without HPSM. Inferential analysis was done using logistic regression to determine the relationship between oral hygiene, caries and HPSM. The logistic regression analysis to determine relationship between caries and HPSM was adjusted for age and sex while the logistic regression analysis to determine relationship oral hygiene and HPSM was adjusted for age. Statistical analysis was conducted using STATA version 12.0. Statistical significance was also established at p values equal to or less than 0.05.

### Standardisation of examiner

Prior to the commencement of the study, one of the authors (TAO) underwent a series of calibration exercises. The first included calibration on the diagnosis of MIH and HPSM. This was done using a coloured picture chart of MIH- and HPSM-affected teeth, with varying degrees of severity of the lesion. This was then followed by the use of live patients with MIH and HPSM. The lesions were graded at different occasions into mild, moderate and severe, according to the criteria earlier stated. The result was coded and fed into the computer. The data were then subjected to a Cohen’s weighted and weighted kappa scores analysis, to determine intra-examiner variability. The intra-examiner variability score for MIH was 0.90 while the intra-examiner variability score for HPSM was 0.85.

## Results

Table 1 shows the socio-demographic profile of the study participants. Out of the 469 pupils that were recruited for this study, 27 (5.8 %) had HPSM. There was no significant difference in the proportion of males and females that had (63 vs 37 %; *p* = 0.06). There also was no significant difference in the proportion of children who had HPSM from the low, middle and high socioeconomic strata (*p* = 0.67).

**Table 1:** Socio-demographic profile of children with hypomineralisation second primary molar

Hypomineralisation Second Primary Molar					
Variables	Present *n* = 27 *n* (%)	Absent *n* = 442 *n* (%)	Total *N* = 469 *N* (%)	*X* ^*2*^	*P* value
Age (years)					
8	10(37.0)	168(38.0)	178(40.3)	2.86	0.41
9	10(37.0)	105(23.8)	115(26.0)		
10	7(25.9)	169(38.2)	176(39.8)		
Sex					
Female	10(37.0)	204(46.2)	214(45.6)	0.85	0.06
Male	17(63.0)	238(53.8)	255(54.4)		
Socioeconomic status					
High	10(37.0)	188(42.5)	198(42.2)	0.32	0.67
Middle	6(22.2)	90(20.4)	96(20.7)		
Low	11(40.7)	164(37.1)	175(37.3)		

Table 2 shows the co-morbidities associated with HPSM. Children with HPSM also had higher prevalence of caries when compared with children without HPSM (44.4 vs 11.3 %; *p* = 0.001). The dmft of children with HPSM was also significantly higher than the dmft of children without HPSM (0.44 vs 0.12; *p* = 0.001). Also, more children with HPSM had poor oral hygiene when compared with children without HPSM (29.6 vs 12.2 %; *p* = 0.01) Of the 1,876 teeth examined, 73 (3.9 %) were affected by HPSM with the mean number of affected teeth per individuals with HPSM being 2.48 ± (1.12).

**Table 2:** Co-morbidity associated with hypomineralisation second primary molar on the child level

	Hypomineralisation second primary molar				
Co morbidity	Present *n* = 27 *n* (%)	Absent *n* = 442 *n* (%)	Total *N* = 469 *N* (%)	*X* ^*2*^	*P* value
Caries					
Caries present	12 (44.4)	50(11.3)	62 (13.2)		0.001
Caries absent	15 (55.6)	392(88.7)	407 (86.8)	24.30	
Dmft	0.44	0.12	0.20		≤0.001
Oral hygiene					
Good	9(33.3)	223(50.5)	232(49.5)	7.34	0.01
Fair	10(37.0)	165(37.3)	175(37.3)		
Poor	8(29.6)	54(12.2)	62(13.2)		

Table 3 highlights the proportion of teeth affected by HPSM on each jaw and each side of the face. The highest proportion of affected primary second molars were in the upper right quadrant (32.9 %) followed by the primary second molars in the lower right quadrant (23.3 %), and the primary second molar in the upper left (21.9 %) and lower left quadrant (21.9 %) respectively. Most of the teeth affected by HPSM (56.2 %) were found on the right side of the jaw. The upper jaw had more teeth affected by HPSM when compared with the lower jaw though the difference was not significant (54.8 vs 45.2 %; *p* = 0.52).

**Table 3:** Number of hypomineralisation second primary molar teeth on each side of the jaw

Quadrant	Number of teeth affected by HSPM
Upper right	24(32.9 %)
Upper left	16(21.9 %)
Lower left	16(21.9 %)
Lower right	17(23.3 %)
Total	73(100.00 %)

Table 4 highlights the severity of HPSM and association with caries and oral hygiene status. The proportion of respondents with mild, moderate and severe forms of HPSM were 48.2, 7.4 and 44.4 % respectively. All children with severe forms of HPSM had caries. The proportion of children with caries increased with the severity of HPSM. Also, more children with severe forms of HPSM had poor oral hygiene when compared with children with mild forms of HPSM.

**Table 4:** Relationship between HSPM severity, caries and oral hygiene

Variables	Mild HSPM *n* = 13 *n* (%)	Moderate HSPM *n* = 2 *n* (%)	Severe HSPM *n* = 12 *n* (%)
Caries status			
Caries present	2(15.4)	1(50.0)	12(100.0)
Caries absent	11(84.6)	1(50.0)	-
Oral hygiene status			
Good oral hygiene	5(38.5)	1(50.0)	3(25.0)
Fair oral hygiene	5(38.5)	-	5(41.7)
Poor oral hygiene	3(22.0)	1(50.0)	4(33.3)

Table 5 shows the outcome of the logistic regression determining the odds of having caries and poor oral hygiene in study participants who had HPSM. Children with HPSM have increased odds having dental caries (AOR: 6.34; CI: 2.78–14.46; *p* = <0.001) and reduced odds of having good oral hygiene (AOR: 0.32; CI: 0.13–0.78; *p* = 0.01) when compare with children without HPSM. In addition, 21 (77.8 %) children with HPSM also had MIH involving one or more permanent first molars.

**Table 5:** Logistic regression determining the odds of having caries and good oral hygiene in study participants with HSPM

Variables	Unadjusted OR	95 % C.I	*P*-value	Adjusted OR	95 % C.I	*P*-value
Caries						
HSPM absent	1.00	-	-	1.00	-	-
HSPM present	6.27	2.78–14.16	<0.001	6.34	2.78–14.46	<0.001
Good oral hygiene						
HSPM absent	1.00	-	-	1.00	-	-
HSPM present	0.33	0.14–0.79	0.01	0.32	0.13–0.78	0.01

## Discussion

This study showed that a significant proportion of children had HPSM both at the child level and the teeth level. A large number of children with HPSM also had MIH co- morbidity. Children with HPSM had higher odds of having caries and poor oral hygiene when compared with children without HPSM. More children with severe form of HPSM have poor oral hygiene and caries when compare with children with mild and moderate forms of HPSM. A large number of children with HPSM also had MIH.

One of the limitations of this study is the age at which the diagnosis of HPSM was determined. It may also be difficult to verify mineralization disturbances of the primary molars at this age as caries, fillings and tooth-wear often mask the effects of developmental disturbances to enamel effects. The second primary molar may also have been lost for a variety of reasons. This may not only result in under-representation of the prevalence of the lesion. It also may affect the true profiling of the severity of the lesion as many teeth affected by HPSM would have also had post-eruptive broke down resulting from prolonged exposure in the oral cavity and may have been extracted.

A second limitation is that this study is a school-based survey thereby limiting the generalisability of the study outcome to the entire population of children in the study environment since not all children are present in school [28]. Despite the limitations of this study, the findings of the study enrich current knowledge about HPSM by highlighting possible co-morbidities associated with HPSM, and similarity and differences in the presentation of MIH and HPSM. The study findings further reinforce the need to screen children for HSPM: it appears that the risk for MIH is high for children who have HSPM. Also the risk for caries and poor oral hygiene is higher for children with HSPM and the risk seems to increase as the severity of HSPM increases. Screening for HSPM can help with early institution of preventive care for prevention of post-eruptive breakdown of teeth affected by HSPM and for the prevention of caries and poor oral hygiene. Where preventive measures are not promptly instituted, HSPM itself as well as the resulting carious lesion and poor oral hygiene can cause social, psychological, and economic distress resulting from pain and multiple hospital visits [29]. Pain also interrupts the ability to learn, play, eat and sleep [30]. Severe caries contributes to a child failing to thrive [31–34] and may also disrupt the development of the child’s psychological capacity because this affects intellectual development and other social skills provided by the activity [35]. When the prevalence of HSPM in this school-based survey is compared to the prevalence of HSPM a population based survey in the same study location [17], the prevalence of the HSPM in this school-based survey was higher than that of the population based survey. We therefore adduce that school based screening for HSPM can result in the identification of large number of children with HSPM, and prompt management of HSPM before complications sets in. The prevalence of HSPM in this study was similar to reports from Dutch [13] and Iraq [18]. The finding in this study may actually be an under-representation of the prevalence of HSPM in the study population as the study was not powered to determine the prevalence of HSPM; the study was powered to determine the prevalence of MIH. Also, HSPM was studied in children 8 years to 10 years old, a period when early tooth erupters may have exfoliated the second primary molar, or may have lost the teeth affected by HSPM for a number of reasons including caries Howbeit, a prevalence of 5 % for any lesion is of epidemiological significance and needs to be taken seriously.

With the high prevalence of HSPM and MIH in this study population [22], it is important for more research to be conducted in the study location to identify possible aetiological and or predisposing factors for HSPM and MIH especially as the co-existence of both lesions is also very high. Elfrink et al. [36] had earlier shown that children with DMH have 4.4 increased odds of having MIH. A study by Ghamin et al. [37] highlights possible predisposing factors for HPSM. Learning about aetiological and predisposing factors for HSPM/MIH in the study environment would help with the institution of community level preventive measures for the lesions.

The pattern and presentation of HSPM in this study is similar to that of MIH. First, the socio-demographic (age, sex and socio-economic status) of children with HSPM did not differ significantly from children without HSPM; a profile reflective of what is observed with MIH [19, 38]. Second, a higher proportion of children with HSPM had caries and poor oral hygiene when compared with children without HSPM: a finding similar to what was observed when children with MIH were compared with children without MIH [15]. In addition the children with severe form of HSPM presented with poorer oral hygiene and had higher caries experience compare with children with mild and moderate form of HSPM. The findings corroborates the postulation by Oyedele et al. [15] that the poorer oral hygiene observed in children with MIH may be a mediating risk factors for the higher prevalence of caries observed in children with MIH. However, unlike MIH where the upper jaw and the left side of the face had more teeth affected [21], children with HSPM in this study did not present with any form of antimere. It may be very important to understand why this distinguishing feature especially with the concerns that HSPM may be a predictor of MIH [36].

Finally, for a sample size this large, it would be expected that atypical restorations/extractions owing to hypomineralisation should be observed. However, prior reports had highlighted that children in the study population rarely report for restorative treatment. The prevalence of unrestored carious lesions are usually very high [18] and treatment are often sought for curative purposes [39]. Screening children for HSPM and providing treatment to prevent post-eruptive breakdown would be a very useful public oral health measure for the study population.

## Conclusion

This study demonstrates that a significant proportion of school children have HSPM and HSPM/MIH co-existence. The morbidities associated with HSPM are significant and can affect the quality of life of children with the lesion negatively. Therefore adequate attention should be given to these children so as to give prompt diagnosis and management of HSPM in order to reduce these co-morbidities. Screening of school children in the study location for HSPM/MIH would be a welcome development.

## Abbreviations

DMH, deciduous molar hypomineralisation; HSPM, hypomineralisation second primary molar; MIH, molar-incisor hypomineralisation
